# Increased Cell Survival of Human Primary Conjunctival Stem Cells in Dimethyl Sulfoxide-Based Cryopreservation Media

**DOI:** 10.1089/bio.2020.0091

**Published:** 2021-02-09

**Authors:** Arianne J.H. van Velthoven, Marina Bertolin, Vanessa Barbaro, Mireille M.J.P.E. Sthijns, Rudy M.M.A. Nuijts, Vanessa L.S. LaPointe, Mor M. Dickman, Stefano Ferrari

**Affiliations:** ^1^ University Eye Clinic Maastricht, Maastricht University Medical Center+, Maastricht, the Netherlands.; ^2^ Department of Cell Biology-Inspired Tissue Engineering, MERLN Institute for Technology-Inspired Regenerative Medicine, Maastricht University, Maastricht, the Netherlands.; ^3^ Veneto Eye Bank Foundation, Venice, Italy.

**Keywords:** ocular surface epithelium, conjunctiva, cell-based therapy, cryopreservation, regeneration

## Abstract

Glycerol and dimethyl sulfoxide (DMSO) are widely used cryoprotectants for freezing human cell cultures. During the manufacturing process of ocular stem cell-based autographs, *ex vivo* cultivated ocular cells are cryopreserved for quality control purposes in accordance with regulatory requirements. The efficiency of the cryopreservation methods is limited by their effect on cell survival and quality. We compared two cryopreservation reagents, glycerol and DMSO, for their influence on the survival and quality of human primary conjunctival cultures. We found increased cell viability after cryopreservation in DMSO compared to cryopreservation in glycerol. The clonogenic and proliferative capacity was unaffected by the cryopreservation reagents, as shown by the colony forming efficiency and cumulative cell doubling. Importantly, the percentage of p63α- and keratin 19 (K19)-positive cells following cryopreservation in DMSO or glycerol was comparable. Taken together, our results demonstrate that cryopreservation in DMSO improves cell survival compared to cryopreservation in glycerol, with no subsequent effect on cell proliferative-, clonogenic-, or differentiation capacity. Therefore, we advise the use of a 10% DMSO-based cryopreservation medium for the cryopreservation of human primary conjunctival cells, as it will improve the number of cells available for the manufacturing of conjunctival stem cell-based autografts for clinical use.

## Introduction

The ocular surface is lined by conjunctival cells covering the sclera and by transparent corneal epithelium covering the stroma. Like most stratified epithelial tissue, the conjunctival and corneal epithelia are constantly regenerated by somatic stem cells.^1^ Disruption of homeostasis in the ocular surface leads to clinical disorders, as seen with pterygia and chemical and thermal burns. Stem cell therapy has proven to be a valuable therapeutic tool for restoring the ocular surface.^2–4^ Autologous tissue-engineered epithelial sheets have recently been approved in Europe for surface reconstruction in patients suffering from limbal stem cell deficiency. For this reason, tissue-engineered conjunctival autografts have been proposed as a treatment for patients with severe conjunctival defects.^5,6^

Primary conjunctival cells for autografts are harvested from autologous ocular biopsies or cadaveric donor tissue from an eye bank.^7–10^ *Ex vivo* cultivation enables the expansion of cells from small donor biopsies without the need for large autografts. However, passaging reduces the proliferative capacity and percentage of p63-positive cells in culture, both of which are stem cell properties associated with clinical success.^2,3,10,11^ To overcome this, the cells are cryopreserved in the presence of a cryoprotectant for long-time preservation and the maintenance of their stem cell properties.^11^

Cryoprotectants prevent cell damage during preservation by reducing cell dehydration and intracellular ice formation.^12,13^ The most widely used cryoprotectants are glycerol and dimethyl sulfoxide (DMSO). Their effectiveness varies in different species and cell types.^14^ DMSO is the standard cryopreservation reagent for biobanking several human cell types, including several pluripotent stem cells and progenitor stem cells.^14–18^ Cryopreservation of human conjunctival cells in 10% DMSO resulted in no difference in their proliferative capacity and expression of progenitor markers compared to cells that were not cryopreserved.^11^ Conversely, the cryopreservation of cultivated rabbit conjunctival cells in 10% glycerol resulted in a higher cell viability than cryopreservation in 10% DMSO.^19–21^

The effect of cryopreservation in 10% glycerol or 10% DMSO on human cultivated conjunctival cells remains to be elucidated. In this study, we aimed to determine cell viability and quality, including proliferative-, clonogenic-, or differentiation capacity, after cryopreservation of human primary conjunctival cells in 10% glycerol or 10% DMSO. Through post-thaw cell viability assays and quality control assays, including expression of phenotypic markers, colony forming efficiency (CFE), and cumulative cell doubling (CCD) assays during an *in vitro* life span test, we found increased viability following cryopreservation in DMSO compared to glycerol and unchanged cell quality. The optimized cryopreservation of human primary conjunctival cells can improve the manufacturing process of stem cell-based transplants.

## Materials and Methods

### Cell culture media

The culture medium was previously described^10^ and consisted of 2:1 Dulbecco's Modified Eagle's Medium and HAM F12 (F12) supplemented with 10% qualified fetal bovine serum (FBS) gamma irradiated, 4 mM l-glutamine, 50 μg/mL penicillin–streptomycin (all from Gibco), 5 μg/mL insulin (Humulin R; Lilly), 0.4 μg/mL hydrocortisone (Merck), 0.18 mM adenine (Merck), 8.1 μg/mL cholera toxin (Sigma-Aldrich), 2 nM triiodothyronine (Liotir), and 10 ng/mL epidermal growth factor (AMSBIO).

### Conjunctival cell culture

Human conjunctival biopsies from corneoscleral buttons of cadaveric donors (ages ranging from 36 to 79 years) were isolated after signed informed consent forms were obtained from the donor's next of kin. Donor biopsies were harvested within 24 hours after death, and all the biopsies were harvested from the inferior fornix area. Human conjunctival cells were cultured as previously described.^3,10^ In short, the cells were isolated from human conjunctival biopsies from three different donors (*N* = 3) by 0.05% trypsin/0.01% EDTA (Gibco) treatment at 37°C and plated on a feeder layer of lethally irradiated 3T3-J2 fibroblasts (40,000 cells/cm^2^). The 3T3-J2 fibroblast immortalized cell line is a kind gift of Prof. Howard Green.^22^ When confluent, 200,000–400,000 cells were cryopreserved in either 0.5 mL CryoStor CS10 (STEMCELL Technologies) or 0.5 mL 10% glycerol freezing solution in cell culture medium, using an isopropanol freezing container for slow rate-controlled cooling (Mr. Frosty, Nalgene). CryoStor CS10 is a serum-free cryopreservation medium containing 10% DMSO. The cells were first stored for 24 hours at 20°C, then at liquid nitrogen temperature for 30 days. After rapid thawing at 37°C, the cells were diluted in culture medium. Next, the cells were centrifuged at 112 × *g* for 5 minutes, and the pellet was resuspended in culture medium. Directly after thawing, cell viability was assessed using a 1:1 0.4% trypan blue staining (Sigma-Aldrich) on a hemocytometer by two independent reviewers, and several *in vitro* quality tests were performed during a complete life span (serial cultivation until senescence). For serial propagation, subconfluent cultures were passaged by 0.05% trypsin/0.01% EDTA (Gibco) treatment at a density of 15,000 cells/cm^2^. For the CCD, the number of cell doublings was calculated at every passage as previously described.^3^

### Colony forming efficiency

The CFE was assessed by plating 1000 viable cells, determined by trypan blue staining, on a feeder layer in a 100 mm culture dish. After 12 days in culture, the cells were stained with a 1:100 crystal violet solution in distilled water (Sigma-Aldrich) and scored as abortive or clonogenic colonies, as previously described.^1,23^ Only colonies containing at least 50 cells were included. The percentage of colony forming cells was calculated by dividing the number of colonies formed by the number of cells plated. For every primary cell culture (*N* = 3), two CFE dishes were plated and analyzed (*n* = 2).

### Immunohistochemistry

At every passage, 10,000–15,000 dissociated cells were loaded per microscope slide using a Cytospin 4 machine (Shandon, Thermo Scientific). After overnight fixation with 4% paraformaldehyde (Santa Cruz Biotechnology) in 1 × phosphate buffered saline (PBS; Gibco) at 4°C, the samples were permeabilized for 30 minutes using 0.5% Triton X-100 (Sigma-Aldrich) solution and blocked for 10 minutes in 1% bovine serum albumin (Sigma-Aldrich) in 1 × PBS (Gibco). The slides were incubated in a primary antibody overnight at 4°C and a secondary antibody in 0.5% bovine serum albumin (Sigma-Aldrich) in 1 × PBS (Gibco) for 1 hour at ambient temperature. The following antibodies were used: rabbit anti-p63-α (1:100;4892; Cell Signaling Technology), mouse anti-Cytokeratin 19 (1:100;ab9221; Abcam), Alexa Fluor-488 goat anti-rabbit IgG (1:500;A11008; Invitrogen), and Rhodamine Red-X goat anti-mouse IgG (1:500; R6393; Invitrogen). The slides were mounted using DAPI Fluoromount-G (Thermo Scientific), and images were captured using a Nikon Eclipse Ti microscope. The quantification of bright p63-positive cells and keratin 19 (K19)-positive cells was automatically performed with Fiji ImageJ^24^ by applying multiple algorithms in sequence, including rolling ball background subtraction, threshold, and particle analysis. For every primary cell culture (*N* = 3), two microscope slides were analyzed (*n* = 2).

### Statistical analysis

All data management and analyses were carried out using SPSS for Windows, version 24.0 (IBM Corp.). The statistical significance between primary cell cultures cryopreserved in glycerol or DMSO was evaluated by a two-tailed Student's t-test. The values were reported as an average of three different primary cell cultures (± standard deviation, SD). Two-sided *p*-values <0.05 (*) and <0.01 (**) were considered statistically significant.

## Results

### Cell survival

**FIG. 1.**
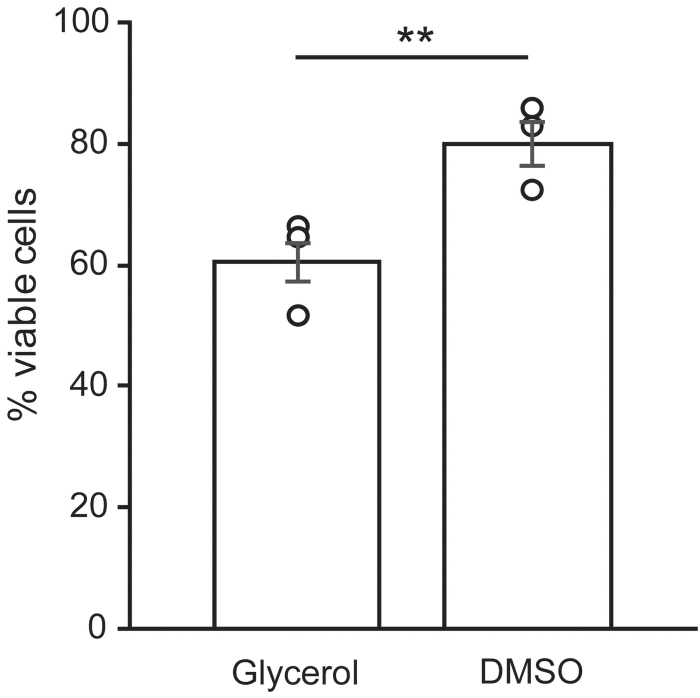
Higher cell viability after cryopreservation in DMSO. Cell survival was assessed directly after thawing using a trypan blue staining. A higher percentage of viable cells was obtained following cryopreservation in DMSO compared to cryopreservation in glycerol (two-tailed *t*-test, **p = 0.001). *Dots* represent the average percentage of viable cells per primary cell culture (*N* = 3). Bars represent the average percentage (±SD) of viable cells. DMSO, dimethyl sulfoxide; SD, standard deviation.

Human conjunctival cells were isolated from conjunctival tissue as previously described^10^ and cultivated until confluent, after which they were cryopreserved in a glycerol-based freezing medium or DMSO-based freezing medium. The percentage of viable cells was assessed using trypan blue staining directly after thawing. Cell counting showed a statistically significant difference between cells cryopreserved in glycerol and DMSO (Fig. 1). The average percentage of viable cells after cryopreservation with DMSO was 79.9% ± 7.0% (±SD), which was significantly higher than the average percentage of viable cells after cryopreservation in glycerol, which was 60.6% ± 7.9% (±SD; *N* = 3; *p* = 0.001). This indicated that the survival of human conjunctival cultures can be improved by cryopreservation in DMSO.

### Proliferative capacity

**FIG. 2.**
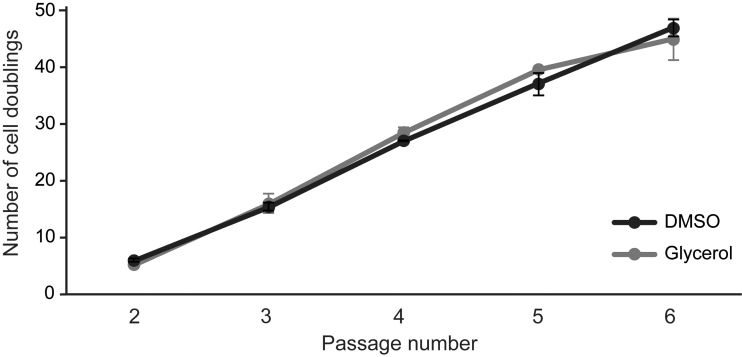
No statistically significant difference in the cell division rate after cryopreservation in glycerol compared to DMSO. The cumulative cell doubling of every passage during a complete life span was assessed. The *dots* represent the average number of cell doublings (±SD) over three different primary cultures (*N* = 3). No significant difference between cells cryopreserved in glycerol and DMSO was observed (two-tailed *t*-test, *p* ≥ 0.05).

Beyond viability, it is important that the cryopreservation media does not affect the proliferative capacity of the conjunctival stem cells. To assess this, we analyzed the CCD of conjunctival cells cryopreserved in glycerol or DMSO. The average CCD showed no statistically significant difference between cells cryopreserved in glycerol or DMSO at every passage (*N* = 3; passage 2: *p* = 0.16, passage 3: *p* = 0.35, passage 4: *p* = 0.21, passage 5: *p* = 0.28, passage 6: *p* = 0.42) (Fig. 2). The total number of cell divisions following cryopreservation in glycerol or DMSO was 45 ± 7.6 and 47 ± 3.3 (±SD) times, respectively. In addition, the time needed to reach confluence and the number of passages until senescence were six passages for both conditions (*N* = 3). Taken together, these data suggest that the proliferative capacity was unaffected after cryopreservation in either glycerol or DMSO.

### Clonogenic capacity

**FIG. 3.**
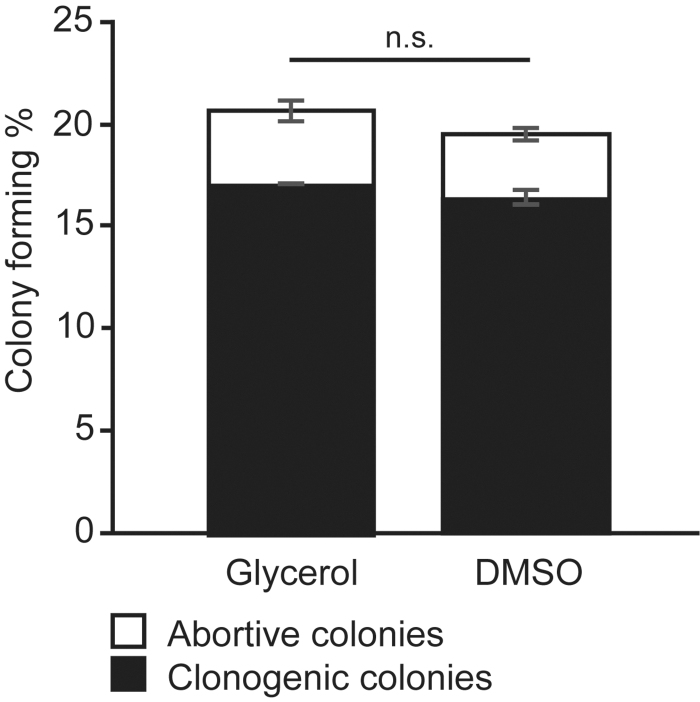
No statistically significant difference in the CFE after cryopreservation in glycerol or DMSO. Per dish, 1000 viable cells were plated and cultured for 12 days. The percentage of abortive and clonogenic colonies was assessed. Bars represent the average percentage (±SD) of colonies from three different cell cultures (*N* = 3, *n* = 2). No statistically significant difference (n.s.) was observed in CFE directly after thawing cells cryopreserved in glycerol or DMSO (two tailed *t*-test, *p* = 0.250). CFE, colony forming efficiency.

In addition to the proliferative capacity, we assessed the post-thaw clonogenic capacity of conjunctival cells cryopreserved in glycerol and DMSO. The clonogenic capacity was assessed by the CFE assay, which refers to the percentage of cells inoculated at a low density that gives rise to colonies. Cells cryopreserved in glycerol or DMSO showed no statistically significant difference in CFE (Fig. 3) (*N* = 3, *n* = 2; *p* = 0.231). The average percentage of abortive colonies and clonogenic colonies after cryopreservation in glycerol was 3.6% ± 1.2% (27 ± 18 colonies) and 17.0% ± 0.1% (117 + 91 colonies; ±SD), respectively. The average percentage of abortive colonies and clonogenic colonies after cryopreservation in DMSO was 3.2% ± 0.8% (26 ± 12 colonies) and 16.4% ± 0.9% (116 ± 84 colonies; ±SD), respectively. These data suggest that the clonogenic capacity of conjunctival cells following cryopreservation in glycerol or DMSO was comparable.

### Cell differentiation capacity

**FIG. 4.**
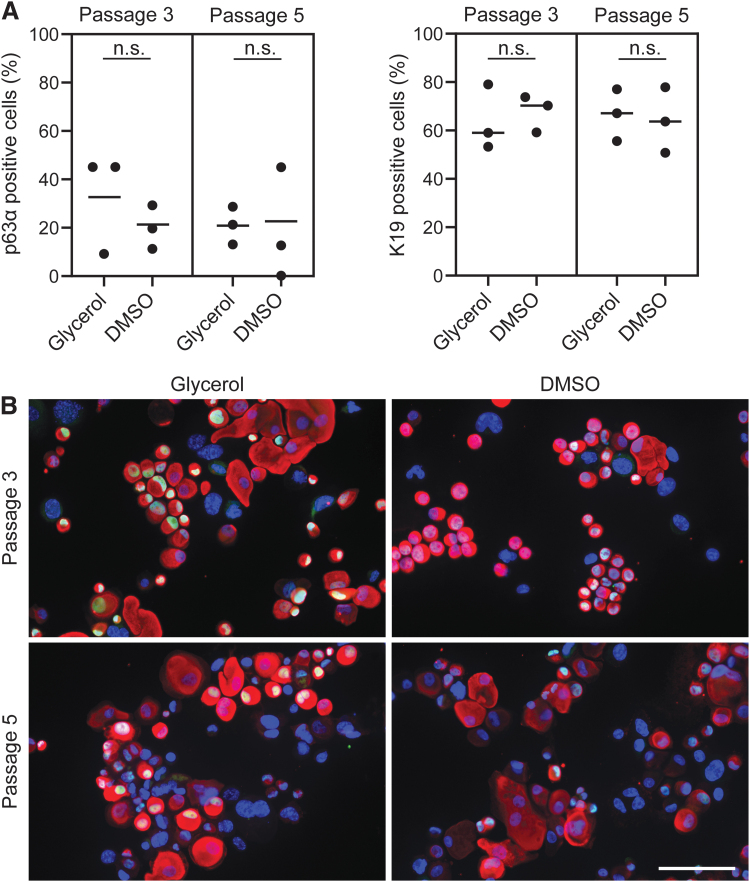
No statistically significant difference in the percentage of cells expressing p63α and K19. **(A)** Quantitative analysis of immunostainings of single cells. There was no statistically significant difference in the mean percentages of cells expressing p63α or K19 after glycerol or DMSO cryopreservation measured after passage 3 or 5 (two-tailed *t*-test, *p* ≥ 0.05). *Dots* represent the average percentage of cells per donor primary cell culture (*N* = 3, *n* = 2). **(B)** Representative immunofluorescence images of K19 (*red*), p63α (*green*), and DAPI (*blue*). Scale bar is 100 μm. K19, keratin 19.

Conjunctival stem cells expressing p63α can differentiate into conjunctival cells expressing K19. The expression of this phenotypic marker assessed by immunofluorescence analysis was not statistically different in the percentage of bright p63α-positive cells (*N* = 3, *n* = 2; passage 3: *p* = 0.42 and passage 5: *p* = 0.87) or K19-positive cells (*N* = 3, *n* = 2; passage 3: *p* = 0.62 and passage 5: *p* = 0.30) following cryopreservation in glycerol or DMSO (Fig. 4). The average percentage of bright p63α cells cryopreserved in glycerol and DMSO was 33.1% ± 21.7% (142 ± 84 cells) and 20.1% ± 11.4% (60 ± 14 cells; ±SD) at passage 3 and 21.0% ± 7.0% (93 ± 25 cells) and 22.9% ± 21.7% (45 ± 51 cells; ±SD) at passage 5, respectively. The average percentage of K19-positive cells cryopreserved in glycerol and DMSO was 63.8% ± 17.9% (318 ± 113 cells) and 68.9% ± 10.1% (245 ± 23 cells; ±SD) at passage 3 and 65.5% ± 12.3% (313 ± 77 cells) and 64.0% ± 12.5% (202 ± 113 cells; ±SD) at passage 5, respectively. These data suggested that the differentiation capacity of conjunctival cells after cryopreservation in glycerol or DMSO was comparable.

## Discussion

The aim of our study was to compare cell viability, phenotype, and clonogenic/proliferative capacity after cryopreservation in glycerol and DMSO as the freezing reagent. To achieve this, we investigated post-thaw cell viability, CFE, CCD, and expression of phenotypic markers.

The main aim of cryopreservation is to preserve biological samples for a prolonged period. Cryopreservation uses very low temperatures to preserve the structure and viability of living cells and tissues, and cryoprotectants prevent cell damage by reducing cell dehydration and intracellular ice formation.^12,13^ To be effective, cryoprotectants must be able to protect and preserve biological structure and function.

With respect to the manufacturing process of autologous tissue-engineered epithelial sheets, including *ex vivo* expanded limbal epithelial stem cells, the European Medicines Agency (EMA) requires the expanded cell suspension to be cryopreserved for quality control purposes before active substance release.^25^ Cryopreservation of primary cells allows testing for microbial contaminants and phenotypic marker expression (e.g., ΔNp63α) analogous to the manufacturing process and quality control of medicines. This approach will likely be adopted by competent authorities for *ex vivo* cultured conjunctival stem cells for the treatment of severe conjunctival defects in the clinic. Anticipating this, selecting a cryopreservation technique that increases cell survival is desirable to improve the availability of stem cells for clinical applications.

In this study, we tested 10% glycerol and 10% DMSO as cryoprotective agents. Glycerol has been used since 1949^26^ and while DMSO is known to enter cells more rapidly, glycerol is less toxic and is thus often used for the preservation of sensitive cells. Previously, cryopreservation of conjunctival epithelial cells in 10% DMSO and 20% FBS showed no effect on the cell quality.^11^ Cryopreservation using CryoStor CS10 has been previously described with promising results for the cryopreservation of umbilical cord blood stem cells and bone marrow-derived mesenchymal stem cells.^27,28^ We have chosen CryoStor CS10 because it provides several advantages, including being animal-product free, reduced processing time, and the consistency of the formulation. A serum-free cryopreservation method is highly important according to guidelines for good manufacturing practice and would improve the safety of an advanced therapy medicinal product.

Our results show that cryopreservation in DMSO leads to higher cell viability compared to glycerol. Cell viability is of high importance for the manufacturing process of stem cell transplants generated from biopsies because it improves the availability of conjunctival stem cells after cryopreservation. This is especially true for autologous transplantation and for the preparation of allografts using biopsies from a living donor, where the number of cells is limited. Optimal cryopreservation conditions could improve the manufacturing process of stem cell-based therapies and reduce the size of the donor biopsies required for cell expansion. An optimized cryoprotectant will also reduce cell loss during cryopreservation.

Regarding proliferation, we measured the colony forming capacity and found no changes in multiplication capacity between cells cryopreserved in glycerol or DMSO. Colony forming capacity has been shown to be important for clinical success.^23^ Keratinocytes with a high proliferative capacity, called holoclones, are used for stem cell-based therapies.^1^ Our findings show a normal clonal level in comparison with previous studies^10,21^ and indicate that the proliferative capacity of the conjunctival stem cells is not altered by cryopreservation. Our findings are consistent with previous studies^1,10^ and indicate that cryopreservation in glycerol and DMSO does not alter proliferative capacity.

We also investigated the expression of p63α, an epithelial stem cell marker, and K19, a conjunctival marker, at passages 3 and 5.^29,30^ We found no changes in expression between cells frozen in glycerol or DMSO. This indicates that the differentiation capacity during culture passaging is unchanged. Clinical stem cell facilities that prepare the stem cell grafts for limbal stem cell therapies are required to freeze down cells and perform quality control tests before transplantation is planned. One of the standard quality tests performed is the assessment of the percentage of p63-positive cells due to its correlation with clinical success.^2^ We showed that conjunctival cells retain a low passage number and preserve the high number of p63-positive cells for transplantation following cryopreservation in either glycerol or DMSO.

One of the limitations of providing tissue for stem cell-based therapies is the cell survival following cryopreservation, which can be improved using an optimal cryoprotectant. Overall, our study showed that primary human conjunctival cells survive cryopreservation better in DMSO than glycerol and that the cell phenotype and other key characteristics were unaffected. Adopting this method in the manufacturing process of autologous tissue engineered epithelial sheets for stem cell-based regenerative medicine could improve their therapeutic availability.
